# S16 National initiatives and cross-country collaboration for HEPA promotion: showcasing experiences of the EU Physical Activity Country Focal Points Network

**DOI:** 10.1093/eurpub/ckae114.269

**Published:** 2024-09-26

**Authors:** Kremlin Wickramasinghe, Catherine Woods

**Affiliations:** University of Limerick, Physical Activity for Health Research Centre, Ireland

## Abstract

The promotion of health-enhancing physical activity (HEPA) within the European Union (EU) requires innovative strategies that can be developed through collaboration across Member States and with strong alignment between research and policy. The EU Physical Activity Country Focal Points Network has been pivotal in fostering such collaborations since its establishment in 2014. The Network has enabled Member States to share best practices, collaborate on projects, and enhance their national HEPA promotion strategies by disseminating the latest research. This symposium will highlight national good practice examples and include a ‘marketplace’ where each country focal point will be available to discuss their national priorities for HEPA promotion with researchers. The symposium will aim to provide a forum to support collaboration between HEPA practitioners, policy makers and researchers, to improve alignment of research with country priorities and identify new opportunities for HEPA promotion and future research areas.

